# Topography of Gng2- and NetrinG2-Expression Suggests an Insular Origin of the Human Claustrum

**DOI:** 10.1371/journal.pone.0044745

**Published:** 2012-09-05

**Authors:** Andrea Pirone, Bruno Cozzi, Larry Edelstein, Antonella Peruffo, Carla Lenzi, Francesca Quilici, Rita Antonini, Maura Castagna

**Affiliations:** 1 Department of Physiological Science, University of Pisa, Pisa, Italy; 2 Department of Comparative Biomedicine and Food Science, University of Padova, Legnaro (PD), Italy; 3 P.O. Box 2316, Del Mar, California, United States of America; 4 Department of Surgery, University of Pisa, Pisa, Italy; University of South Florida College of Medicine, United States of America

## Abstract

The claustrum has been described in the forebrain of all mammals studied so far. It has been suggested that the claustrum plays a role in the integration of multisensory information: however, its detailed structure and function remain enigmatic. The human claustrum is a thin, irregular, sheet of grey matter located between the inner surface of the insular cortex and the outer surface of the putamen. Recently, the G-protein gamma2 subunit (Gng2) was proposed as a specific claustrum marker in the rat, and used to better delineate its anatomical boundaries and connections. Additional claustral markers proposed in mammals include Netrin-G2 in the monkey and latexin in the cat. Here we report the expression and distribution of Gng2 and Netrin-G2 in human post-mortem samples of the claustrum and adjacent structures. Gng2 immunoreactivity was detected in the neuropil of the claustrum and of the insular cortex but not in the putamen. A faint labelling was present also in the external and extreme capsules. Double-labelling experiments indicate that Gng2 is also expressed in glial cells. Netrin-G2 labelling was seen in neuronal cell bodies throughout the claustrum and the insular cortex but not in the medially adjacent putamen. No latexin immunoreactive element was detected in the claustrum or adjacent structures. Our results confirm that both the Gng2 and the Netrin-G2 proteins show an affinity to the claustrum and related formations also in the human brain. The presence of Gng2 and Netrin-G2 immunoreactive elements in the insular cortex, but not in the putamen, suggests a possible common ontogeny of the claustrum and insula.

## Introduction

The claustrum is a subcortical nucleus present in all mammalian species examined so far. The human claustrum is a symmetrical, thin, and irregular sheet of grey matter. It lies between the inner surface of the insular cortex and the outer surface of the putamen, separated by them by the extreme and external capsule, respectively [1]. Anatomically (but not functionally) the claustrum may be subdivided into two parts [2]: *a*) the dorsal part, or insular claustrum, located below the insular cortex; and *b*) the ventral part, or temporal claustrum, placed below the piriform cortex [3], [4].

In the mammalian brain, the claustrum is reciprocally and diffusely connected to the cerebral cortex [5], [6], [7], [8]. The presence of claustro-cortical connections has not been demonstrated in the human brain, but their presence is implicit [9]. Although the functional significance of the human claustrum remains unknown, its position and neural connections suggest a possible role in the integration of the information that underlies conscious perception [10].

The ontogenetic origin of this enigmatic structure has been a matter of debate. Some authors suggested that the claustrum shares a common origin with the putamen, others, in contrast, considered the claustrum analogous to the cortex, and possibly a part of it. A third faction speculate that it has both a pallial and subpallial origin [11].

The search for a claustrum-specific protein has drawn considerable the attention and a few molecules were proposed as candidates. The G-protein gamma2 subunit (Gng2) belongs to the subfamily II of the γ subunits. This protein plays a key role in signal transduction systems where receptors are coupled to heterotrimeric G proteins [12]. In a recent study, Gng2 was identified as a specific rat claustrum marker, and thus used as a tool to better delineate its anatomical boundaries and connections [13]. Netrin-G2 belongs to the UNC6/netrin family; the netrins are secreted molecules which regulate axon development [14]. In a former study, by in situ hybridization, a strong expression of Netrin-G2 has been reported in the monkey claustrum [15]. Furthermore, latexin, an endogenous inhibitor of the A/B subfamily of metallocarboxypeptidases has been described as a specific marker of the claustrum and selected areas of the cortex in the cat [16].

To the best of our knowledge, there are no reports on the Gng2, Netrin-G2 and latexin immunoreactivity (-ir) distribution in the human claustrum. Therefore, the present study is aimed at evaluating the presence of these proteins in post-mortem samples of the human claustrum and adjacent structures, to assess whether they could be considered claustrum markers also in the human, and to evaluate the possible relationship with the putamen and/or the adjacent cortex.

## Materials and Methods

### Tissue samples

In this study we used archival samples obtained from seven patients of different sex and age, with no history of psychiatric or neurological disorder. The average age was 59.6 years and the average post-mortem delay was 26 h.

**Figure 1:**
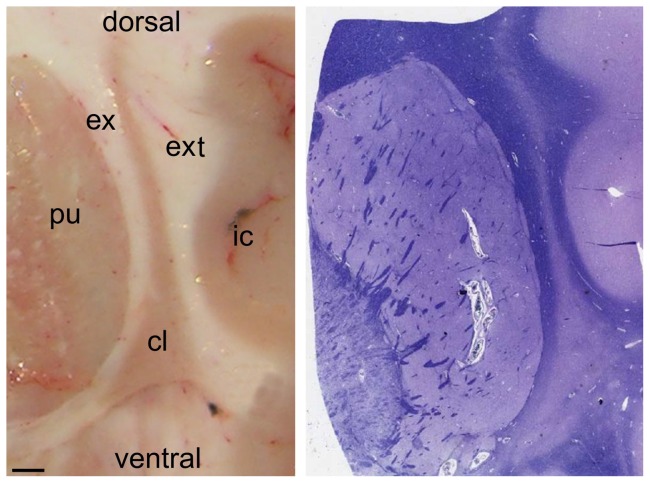
Claustrum localization in the brain. Photographs of a coronal section of a post-mortem specimen (left) and a Luxol fast blue stained section (right) showing the anatomical correlation between the claustrum (cl) and the adjacent structures. pu, putamen; ic, insula cortex; ex, external capsule; ext, extreme capsule. Scale bar, 1 mm.

**Figure 2:**
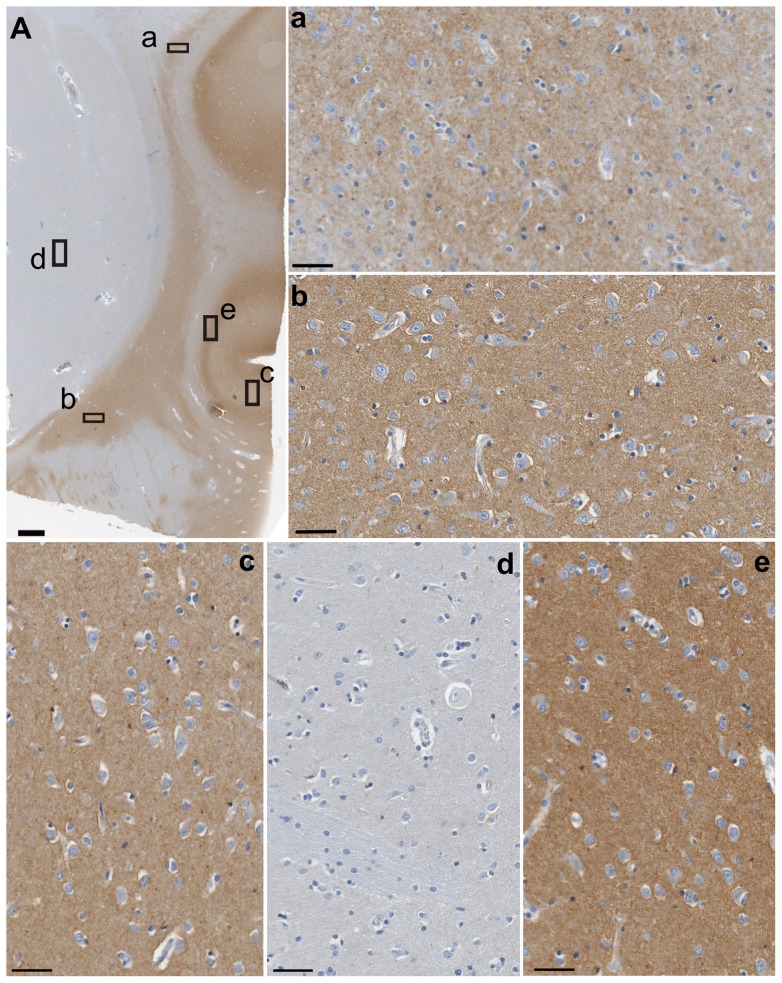
Gng2-ir distribution in the claustrum and adjacent structures. (A) Low magnification image. (a–e) Higher magnification images of A correspond to the dorsal claustrum (a), the ventral claustrum (b), the insular cortex (c), the putamen (d) and the VI layer of the insula (e). Scale bar  = 1 mm (A), 50 µm (a–e).

**Figure 3:**
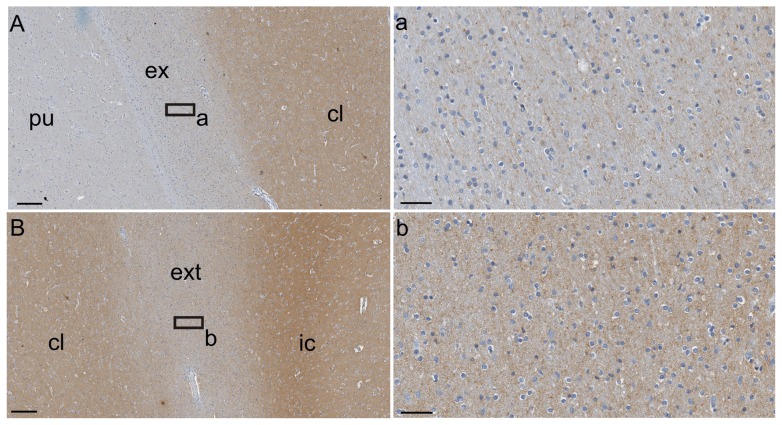
Gng2-ir distribution in the capsules. (A, B) Low magnification images. (a, b) Higher magnification images of A and B correspond to (a) the external capsule (ex) and (b) to the extreme capsule (ext). pu, putamen; ic, insular cortex; cl, claustrum. Scale bar  = 200 µm (A, B), 50 µm (a, b).

The samples consisted of blocks approx 5 cm thick, including both the insular and temporal subunits of the claustrum, surrounded by portions of the adjoining structures (extreme and external capsules, insular cortex, putamen). The samples were carefully dissected during post-mortem procedures performed by qualified pathologists at the S. Chiara Hospital, University of Pisa. The brain samples were removed for routine diagnostic scopes, following a procedure approved by the Ethic Committee of the University of Pisa (protocol number 3482).

The blocks were fixed by immersion in buffered formalin, washed in phosphate saline buffer (PBS) 0.1 M, pH 7.4, processed for paraffin embedding, sectioned at a thickness of 3 μm and mounted on positively charged slides. Epitope retrieval was carried out at 120°C in a pressure cooker for 5 min. All tissue blocks were cut in the coronal plane.

**Figure 4:**
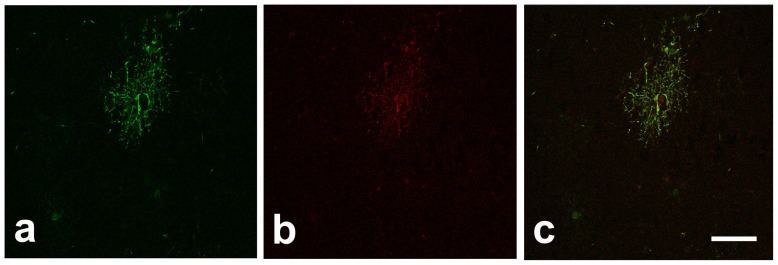
Gng2 are expressed in glial cells as analyzed by double-labeling immunohistochemistry and confocal microscopy. Gng2 identified by anti-rabbit antiserum conjugated with FITC (a), GFAP identified by anti-mouse antiserum conjugated with TRITC (b); merge image (c) shows co-localization of the two substances. Scale bar  = 10 µm.

**Figure 5:**
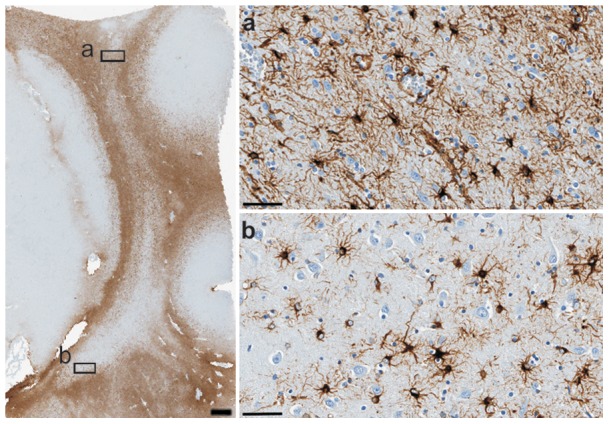
GFAP-ir distribution in the claustrum and adjacent structures. (A) Low magnification image. (a, b) Higher magnification images of A correspond to the dorsal claustrum (a), the ventral claustrum (b). Scale bar  = 1 mm (A), 50 µm (a, b).

To better localize the claustrum boundaries, some sections were stained with the Luxol Fast Blue method.

### Immunohistochemistry

A rabbit polyclonal anti-Gng2 antibody (Sigma-Aldrich, HPA003534, Lot. Number A00951, Species Reactivity: Human; dilution 1∶100); a mouse monoclonal (4F11) to Netrin-G2 (abcam, ab111994; dilution 1∶250); a rabbit polyclonal to Latexin (abcam, ab115583; dilution 1∶50); and a mouse monoclonal to GFAP (Dako, Milan, Italy, at a 1∶200 dilution) were used in this study. Sections were rinsed in PBS and incubated in 1% H_2_O_2_-PBS for 10 minutes, then to reduce non-specific staining preincubated in PBS with 0.3% Triton X-100 (TX) (Sigma-Aldrich, St Louis, MO, USA) and 5% normal goat serum (for Gng2 and Latexin) (Vector Labs, Burlingame, CA) and in 5% normal horse serum (for Netrin-G2 and GFAP) (Vector Labs, Burlingame, CA). Next, sections were incubated overnight in a humid chamber at 4°C with the primary antibody diluted in PBS with 0.3% TX and 1% normal serum. After several washings in PBS, sections were incubated for 1 hour at room temperature in biotinylated goat anti-rabbit immunoglobulin (for Gng2 and Latexin) and biotinylated horse anti-mouse immunoglobulin (for Netrin-G2 and GFAP) (Vector Labs, Burlingame, CA), diluted 1∶300 in PBS. Sections were then washed for 3x10 minutes in PBS, and incubated for 1 hour at room temperature in avidinbiotin-horseradish peroxidase complex (ABC; Vector Labs, Burlingame, CA), diluted 1∶125 in PBS. After washing for 3x10 minutes in Tris/HCl (pH 7.6), peroxidase activity was detected by incubation in a solution of 0.125 mg/ml diaminobenzidine (Sigma-Aldrich, St. Louis, MO, USA) and 0.1% H_2_O_2_ in the same buffer for 10 minutes.

**Figure 6:**
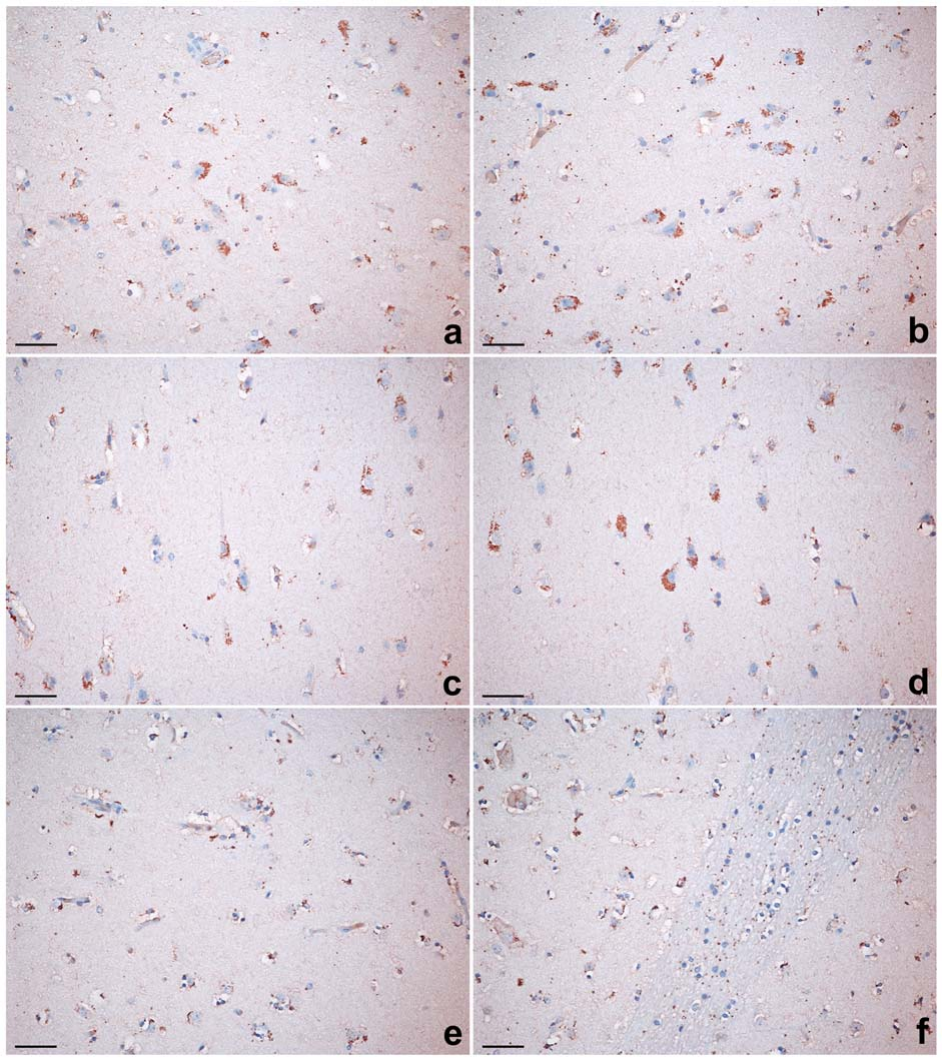
Netrin-G2-ir distribution in the claustrum and adjacent structures. Positive perikarya in the claustrum (a, b) and in the insular cortex (c, d). No immunoreactivity was seen in the putamen (e, f). Scale bars  = 50 µm.

The sections were examined and photographed with a light microscope (Leitz Diaplan) equipped with a Nikon digital camera.

### Double-labelling immunohistochemistry and confocal microscopy

Double staining experiments were performed to check whether Gng2 was co-localized with GFAP and/or Neurofilament-200. Tissue sections were fixed as described above, washed with PBS, blocked by a 30 minute incubation with bovine serum albumin and then incubated overnight at 4°C with the anti-Gng2 antibody described above and with *a*) an anti-GFAP antibody raised in mouse (Dako, Milan, Italy, at a 1∶200 dilution); or *b*) an anti-Neurofilament-200, raised in mouse (Sigma-Aldrich, Milan, Italy, at a 1∶40 dilution). Excess primary antibody was eliminated by rinsing three times in PBS. The sections were then incubated (2 h at room temperature) with secondary fluorescent antibodies against rabbit immunoglobulins-FITC, (Santa Cruz Biotechnology, Santa Cruz, CA, USA, dilution 1∶500), and goat anti-mouse immunoglobulins-TRITC (DakoCytomation, Glostrup, DK, dilution 1∶500). Afterwards, sections were washed three times in PBS and finally mounted with FlourSave™Reagent (Calbiochem, San Diego, CA, USA). Immunostained slides were examined, and images were obtained using a Leika TCS confocal microscope.

The employed anti-Gng2 antiserum was already validated by the Human Protein Atlas (http://www.proteinatlas.org/ENSG00000186469/summary). Furthermore, in our lab the specificity of the immunohistochemical staining reactions was tested in repeated trials as follows: substitution of either the antibody, the anti-rabbit IgG, or the ABC complex by PBS or non-immune serum. Under these conditions staining was abolished.

## Results

Our data concern both subunits (insular  =  dorsal; ventral  =  temporal) of the claustrum. In all tissue blocks and in Luxol-stained sections, the claustrum appeared as a thin ribbon of grey matter located medial to the insular cortex and lateral to the putamen, separated by the extreme capsule from the former and by the external capsule from the latter, respectively (Fig. 1). Its shape widens in the downward direction and becomes triangular in the temporal part of the structure. Luxol Fast Blue, a widely used specific staining for myelin, allowed us to clearly define the capsules and thus delineate the anatomical boundaries of the claustrum.

At the light microscope level, Gng2 and Netrin-G2 immunostaining identified several positive elements throughout the claustrum and the adjoining structures. On the contrary, no Latexin labelling was detected.

### Gng-2 immunostaining

The distribution of Gng2-ir was limited to the claustrum and to the insular cortex neuropil, but no immunostaining was present in the putamen (Fig. 2A). The density of staining was lower in the dorsal part of the structure (Fig. 2a) and higher in the ventral (Fig. 2b). A faint Gng2-ir signal was detected both in the external and in the extreme capsule (Fig. 3). In the external capsule the immunoreactivity followed the apparent direction of the nerve fibers (Fig. 3a), while in the extreme capsule it was irregular and dispersed among the white matter (Fig. 3b).

In the insular cortex a dense plexus of Gng2-ir was seen in the VI layer (Fig. 2e); the other layers were characterized by a lower staining density (Fig. 2c).

Double-labelling experiments and confocal microscopy showed that Gng2 and GFAP were co-localized in the same elements, characterized by a small body and a rich arborisation of slender processes (Fig. 4). GFAP single labelling (Fig. 5) confirmed that the claustrum areas expressing Gng2 were characterized by the presence of astrocytes.

On the other hand, filament protein N-200 showed no co-localization with Gng2 protein within our experimental setting.

### Netrin-G2 immunostaining

Netrin-G2 labelling was seen in cell bodies throughout the claustrum and the insular cortex but not in the medially adjacent putamen (Fig. 6). In the insular cortex, Netrin-G2-ir neurons were mainly distributed in the V and VI layers, in which they displayed a pyramidal shape with the main axis radial to the pial surface (Fig. 6 c, d). The claustrum showed numerous positive perikarya with a fusiform or oval shape, and the main axis appeared to be tangential to the pial surface (Fig 6 a, b).

### Latexin immunostaining

No latexin immunoreactive element was observed in the examined sections, either in the claustrum or in adjacent structures.

## Discussion

Topography, boundaries and structure of the human claustrum have been described in several papers [17], [18], [19], [20]. The search for a specific claustral marker identified a number of potential candidates. The relevance of a claustrum-specific marker is in that it may help clarify the ontogenetic descent of the structure and identify close-related brain components. In a recent study, the Gng2 protein has been identified as a specific claustrum marker in the rat [13]. Other recently defined claustral indicators include Netrin-G2 in the monkey [15] and latexin in the cat [16]. In the present study we evaluated, for the first time, whether these markers apply also to the human claustrum.

In our experimental series, as in other mammals, the Gng2-ir was localized in the claustrum. However, contrarily to what has been reported in the rat [13], in our investigation Gng2-ir was also present in the insular cortex and to a small extent in the external and extreme capsule. The presence of immunostaining in these structures may indicate that this protein is expressed in insular elements, at least in humans. Several studies performed on non-human primates described connections between the claustrum and many cortical and sub-cortical regions [21], [22], [23], [24]. Based on that, we can speculate that the claustrum could be reciprocally connected to the insular cortex via the extreme capsule.

The original description of Gng2 in the rat claustrum [13] implied that the protein was expressed in neurons, although the resolution of the images was directed to identify relatively large structures and not single cells and no co-localization studies were performed. Our data show that Gng2 is co-localized with GFAP, and therefore expressed by astrocytes, a fact substantiated by the morphology of positive elements observed at the confocal microscope. Failure to co-localize Gng2 with the neurofilament protein N200 further suggest that the protein is present in glial cells. However, given the limits of post-mortem samples, our data cannot exclude the presence of Gng2 also in a population of neurons, as formerly reported in the human cerebral cortex (http://www.proteinatlas.org/ENSG00000186469/normal/cerebralcortex). Further studies, with perfusion fixation performed in rodents, could help solve the issue.

Our findings regarding the Netrin-G2 showed that this marker protein was present in neurons of the claustrum and insular cortex, but not in the medially adjacent putamen. These results were in line with those described in the monkey claustrum where, employing in situ hybridization, a strong expression of Netrin-G2 was observed [15].

Latexin positive neurons were reported to be present in the cat claustrum and insular cortex [16]. In the present study, we detected no latexin-immunoreactive element in the section of the human claustrum and adjacent areas, including the cortex. Possible explanations include species-specific variability or potential loss of signal due to post-mortem interval occurred before sampling. However, we emphasize that latexin mRNA was not detected in the monkey neocortex [25] and the selective value of this protein as a claustrum marker should be further investigated, at least in primates.

In our experimental series, immunostaining with both Gng2 and Netrin-G2 were able to well delineate the structure of the claustrum and its borders. However, in the case of Gng2, the presence of immunostained elements in the adjacent capsules and insular cortex may either indicate a lesser specificity of the protein as marker in the human, or a common ontogenetic origin of all positive formations. To this effect, the findings reported in this article may contribute to an understanding of the ontogeny of this enigmatic structure.

The ontogeny of the claustrum is still open for discussion. Three main theories exist. According to the pallial theory, the claustrum is considered a derivative of insular cortex. The sub-pallial theory depicted the claustrum as derived from the ganglionic eminence or paleostriatal angle via a ventrolateral migration of the ventricular matrix along with the basal ganglia. The third theory, or hybrid theory, supports the hypothesis that the claustrum has both a pallial and a sub-pallial derivation [11]. Gene expression studies in mice demonstrated the presence of pallial markers in the claustrum and in the basal amygdala but not in the striatal structures [26], [27].

Our data provide evidence in support of the pallial theory, because the claustrum and the insular inner layer revealed a very similar Gng2-ir and Netrin-G2 distribution pattern while no immunostaining was detected in the putamen. Our findings also support results obtained in non-human primates, in which the expression of Netrin-G2 is confined in the extreme capsule, in layer 6 of the insular cortex, and in the claustrum [15].

In conclusion, our data contribute to the understanding of the ontogeny of the claustrum and support the theory of a pallial origin of this enigmatic structure in the human.
